# P15 Acupuncture treatment of Costochondritis, a case series

**DOI:** 10.1093/rap/rkac067.015

**Published:** 2022-09-28

**Authors:** Rosemary Alexander

**Affiliations:** Rheumatology Department Royal Free Trust, London, United Kingdom

## Abstract

**Introduction/Background:**

I had been practising acupuncture for 30 years and noticed while working in the rheumatology clinic that costochondritis had a particular response to acupuncture. As this condition is extremely debilitating and often misdiagnosed, a case series of 8 cases is presented, the majority of whom were seen in primary care.

**Description/Method:**

Costochondritis is characterised by anterior chest pain associated with painful tender costochondral (CC) joints felt on palpation. These mainly affect the 2nd to 5th costochondral junctions, either unilateral or bilateral. Other areas of the anterior and lateral chest wall can also be affected. The pain is often severe and nocturnal, of sudden onset and can be associated with shock, nausea and dyspnoea, causing patients to call for an ambulance. Tietze's syndrome is a rare variant of this, usually affecting younger patients.

Patients with persistent chest wall pain were offered acupuncture. Details of age, duration of symptoms, number of treatments and VAS pain scores were recorded day and night, (Table 1) pre and post treatment Patients with thoracic pain were excluded.

0.25*25mm and 0.25*30mm needles were used with silicon tips and a guide tube, to help prevent painful insertion. An infra-red lamp (Bio-lamp) was used + electro-acupuncture (EA) if insufficient improvement after two close treatments, sessions were continued weekly.

Acupuncture points used were local tender/trigger points + distal Chinese points P6 and LI4. Other points used were CV 17, GB 34, SI6, GB39 or Sp 6 and St36. An average of 4.7 sessions were needed.

Audit of Acupuncture Treatment Table 1

Six patients took daily analgesia which was rapidly reduced to nil. All completed their treatment until their pain stabilised. There were no adverse events.

Average improvement in the day was 89% and night 85%. Recurrences of pain improved by 11% in the day and 72% at night. The attacks of severe chest pain and dyspnoea stopped, abating anxiety.

**Discussion/Results:**

Costochondritis is a highly debilitating condition and I had noticed that acupuncture could be a dramatic treatment for this[i]. It can affect up to a third of patients attending emergency departments with atypical chest pain[ii] and 1-3% of patients in primary care[iii].

It seems to be a missed diagnosis by Accident and Emergency (A&E) doctors and GPs, perhaps because the chest wall was not carefully palpated. One patient had ankylosing spondylitis (AS) and another had systemic lupus erythematosus but attributed her pain to prolonged laughter. Five patients attended cardiology clinics including one who had a coronary angiogram. Another had tests for upper abdominal pain which also were normal.

All the patients were female although quoted F:M ratio is 7:3

Differential diagnoses include: acute coronary syndromes, lung problems, trauma, upper gastroenterology pathology or neoplasia

Pathogenesis is unclear but the onset can be acute and follow:

Respiratory tract infection  Extreme sneezing, coughing or laughter

Chest wall trauma        Micro trauma from costovertebral dysfunction

Fibromyalgia          Inflammation from inflammatory joint disease.

[i] Alexander R; White A. Acupuncture in a Rheumatology Clinic. Acupuncture in Medicine; Dec 2000 vol.18 (no.2): 100-103

[ii] Disla E; Rhim HR; Reddy A; Karten I; Taranta A. A prospective analysis in an emergency department setting. Arch Intern Med. 1994; 154(21):2466-2469

[iii] Ayloo A; Cvengros T; Marella S. Evaluation and treatment of musculoskeletal chest pain. Prim Care (Review) Dec 2013 40(4): 863-87

**Key learning points/Conclusion:**

Normal management of costochondritis includes analgesia, physiotherapy, steroid injections and occasionally surgery[i]. 30% can have protracted pain. There are isolated reports of acupuncture for this condition, integrated with conventional medicine[ii] [iii]. Acupuncture also has been used for myofascial trigger points including costochondritis[iv].

Acupuncture is now an accepted modality of pain management and in 2021 was recommended in NICE guidelines for the treatment of Chronic Primary Pain alongside exercise programmes and CBT ahead of analgesic medication[v]. It has also been recommended in the management of low back pain in patients aged 60 years and over as a non-drug treatment[vi].

This paper highlights the fact that acupuncture appears to be a safe effective treatment for costochondritis, with further research and more access needed for this therapeutic modality.

Increased awareness of this condition would make complex invasive investigations less necessary.

[i] Gologorsky R; Hornik B; Velotta J. Surgical Management of Medically Refractory Tietze's Syndrome. Annals of Thoracic Surgery; Dec 2017; vol. 104 (no. 6)

[ii] Jonkman FAM.; Jonkman-Buidin M.L; Stolwijk P.W.J. Surprise after successful treatment of a patient with noncardiac chest pain using acupuncture. Medical Acupuncture; Feb 2015; vol.27(no 1):83-90

[iii] Lin, Katerina; Tung, Cynthia. Integrating Acupuncture for the Management of Costochondritis in Adolescents. Medical Acupuncture; Oct 2017; vol.29(no 5): 327-330

[iv] Baldry P. Cardiac and non-cardiac chest wall pain. Acupuncture in Medicine;1997; vol.15(no.2): 42-45

[v] NICE guideline; Chronic Pain (primary and secondary) in over 16s (NG 193 April 2021)

[vi] Traeger A; Underwood M;Ivers R; Buchbinder R. Low back pain in people aged 60 years and over. BMJ 2022 vol. 376: e066928

**P15 Table 1:** 

PATIENT	AGE	DURATION OF	NUMBER OF	VAS INITIAL (0-10)		VAS FINAL	
	YEARS	PROBLEM	TREATMENTS	DAY	NIGHT	DAY	NIGHT
1	37	5 MTHS	4	10	10	0	0
2	26	18 MTHS	8 (1 EA)	5.5	7.5	0	2
3	65	3 WEEKS	5 (+EA)	7	9	1	1
4	58	4 DAYS	4	9	7	2	2
5	62	10 WEEKS	7 (+EA)	6	8	0	2
6	34	6 WEEKS	4	4	7	2	2
7	28	9 MTHS	3	1	4	0	0
8	25	5 YEARS	3	8	7	0	0
Recurrence of pain							
3	65	1 WEEK	4 (+EA)	8	8	1	2
6	34	5 WEEKS	2	2	8	2	2
7	28	2 WEEKS	1	1	3	1	1

